# The Poisonous Dose of Chloral

**Published:** 1870-08

**Authors:** 


### The Poisonous Dose of Chloral.

We have received the following important note from Dr. J. R. Reynolds:

"I was called to see a lady of middle age, who had, for the relief of neuralgia, taken hydrate of chloral.

"On the third day before my seeing her, she had taken gr. 10 and gr. 15, and had found much relief. On the day before, she had taken a larger dose, with good effect.

"On the day of my being summoned, the dose had been increased to gr. 45 or gr. 50, and there had followed complete relief of pain; but in the course of an hour, some 'faintness' was felt, and when I saw the patient this had increased to an alarming degree. Two hours had passed since the last dose was taken, and I found the patient with cold extremities, an excessively rapid, weak, irregular and intermittent pulse, jactitation of limbs, an intolerable sense of sinking, and oppression at the pit of the stomach; gasping breathing, and confusion of thought.

"I observed at this time, and for three-quarters of an hour subsequently, that the radial, temporal, and tibial pulses were all of the character I now describe—frequent, weak, irregular in both force and rhythm, and frequently intermittent—but that the heart was acting regularly, although with increased frequency and diminished force.

"Stimulants, with white of egg, was administered freely; warmth was applied to the extremities, sinapisms were put on the cardiac region, fresh air was introduced plentifully into the room, and, at the end of an hour from my first seeing the patient, the pulse had become much steadier, though still very frequent and very weak. The syncopal feeling had diminished, the feet were warm, and there was a tendency to sleep.

"This state of comparative freedom from urgently dangerous symptoms lasted for longer than an hour, when—without any apparent cause—they returned with increased severity. The patient now seemed in the gravest danger. The superficial pulses were almost imperceptible; and, when they could be detected, presented the character I have described. Still the heart was regular in its beat, although feeble, and intensely rapid in its pulsations. The mind wandered much; there was utter prostration of muscular strength, the limbs being extended, the head low, and the aspect was, at times, that of impending dissolution. There was a great dyspnœa, a sense of suffocating oppression at the base of the chest (in front), and urgent thirst.

"The treatment previously adopted was again pushed vigorously, and, at the end of an hour and a half, relief was obtained, and sleep followed.

"The next morning I found the pulse quite regular, and of its normal frequency,

"I have written this hastily, but pray put it in your own way, and make any or no use of it, as you think best.

"The points of interest that occurred to me were: 1st, the dose; 2nd, the time between its administration and the appearance of symptoms; 3rd, the recurrence of symptoms after their temporary cessation; 4th, the curious effect on the vessels, which was obviously not due to effect on heart; 5th, the relief by food and stimulant. I found that the albumen (of two eggs) was that which was followed by a calming effect, and a tendency to sleep."—*London Practitioner.*—*Baltimore Reprint.*

